# Prepulse Inhibition and P50 Suppression in Relation to Creativity and Attention: Dispersed Attention Beneficial to Quantitative but Not Qualitative Measures of Divergent Thinking

**DOI:** 10.3389/fpsyt.2022.875398

**Published:** 2022-06-09

**Authors:** Marije Stolte, Bob Oranje, Johannes E. H. Van Luit, Evelyn H. Kroesbergen

**Affiliations:** ^1^Department of Orthopedagogics: Cognitive and Motor Disabilities, Utrecht University, Utrecht, Netherlands; ^2^Center for Neuropsychiatric Schizophrenia Research (CNSR) and Center for Clinical Intervention and Neuropsychiatric Schizophrenia Research (CINS), Mental Health Center Glostrup, Glostrup, Denmark; ^3^Behavioural Science Institute, Radboud University, Nijmegen, Netherlands

**Keywords:** children, sensory gating, sensorimotor gating, ADHD, psychophysiological gating, creativity, divergent thinking

## Abstract

The current study investigated whether lower sensory and sensorimotor gating were related to higher levels of creativity and/or attentional difficulties in a natural population of primary school children (9- to 13-year-old). Gating abilities were measured with P50 suppression and prepulse inhibition of the startle reflex (PPI). The final sample included 65 participants in the P50 analyses and 37 participants in the PPI analyses. Our results showed that children with a high P50 amplitude to testing stimuli scored significantly higher on the divergent outcome measures of fluency and flexibility but not originality compared to children with a lower amplitude. No significant differences were found on any of the creativity measures when the sample was split on average PPI parameters. No significant differences in attention, as measured with a parent questionnaire, were found between children with low or high levels of sensory or sensorimotor gating. The data suggest that quantitative, but not qualitative measures of divergent thinking benefit from lower psychophysiological gating and that attentional difficulties stem from specific instead of general gating deficits. Future studies should take the effect of controlled attention into consideration.

## Introduction

There appear to be both strengths and weaknesses to decreased sensory gating, one of the neurophysiological measures thought to reflect the brain's early filtering mechanism of incoming environmental information to reduce strain on higher brain functions (1). On the one hand, reduced filtering may cause more distractibility and errors due to the flooding of higher brain functions with unnecessary information (2, 3). Hence, overloading the higher order cognitive system, this higher influx of information from the environment could result in attentional problems. Micoulaud-Franchi et al. (4) showed that the experience of being flooded with sensory stimuli that is often reported by individuals with ADHD could be explained by reduced sensory gating, which strengthens previous reports on this topic (5). On the other hand, it can be beneficial during a situation in which there are several courses of action necessary or during creative acts, by giving an individual more information to choose from Carson et al. (6), Gonzalez-Carpio et al. (7), and Zabelina et al. (8). A common definition of creativity is that it is the interaction between process, ability, and environment to create something meaningful and original based on contextual factors (9, 10). Since the benefits of promoting creativity in primary school curricula are increasingly accepted and implemented (11, 12) while in turn creativity has been linked to attentional difficulties (13) the current paper investigated sensory and sensorimotor gating in relation to creativity and attention. Distractibility and inattention are often thought of in a negative connotation due to the social and reward structures that are in place in current western societies (roughly 60% of the general population is thought to have at least one symptom of hyperactivity or inattention) (14), leading to stigma and lower quality of life in those with an attentional disorder (15, 16). The current study aimed to investigate whether there are also benefits to such attentional profiles that are associated to a higher influx of sensory stimuli due to reduced sensory and/or sensorimotor gating.

Two paradigms commonly believed to measure the early, pre-conscious filtering of irrelevant or distracting information are sensorimotor gating (prepulse inhibition of the startle reflex, or PPI) and sensory gating (P50 suppression). During PPI, a muted or inhibited magnitude of the startle reflex is observed when an intense, startle eliciting stimulus is presented after a weak stimulus (the prepulse). In humans, this is usually assessed with electromyography (EMG) of the orbicularis oculi muscle (17–19).

Similar to PPI, P50 suppression represents the influence of inhibitory processes triggered by earlier presented stimuli. In a typical P50 paradigm, participants are presented pairs of identical auditory stimuli with an ISI of 500 ms (8, 20). In healthy participants, the event related potentials (ERPs) to the second stimulus are usually reduced, starting from the positivity emerging in the electroencephalogram (EEG) after 50 ms (P50) onwards (21–23). This decrease in P50 amplitude is thought to reflect sensory gating.

Some studies report that P50 suppression and behavioral attentional measures are positively correlated (24–26), but studies showing that measures of attention and P50 suppression are unrelated also exist (27, 28). This discrepancy is likely due to the different attentional measures that were assessed in these studies. In children (age 9–14), symptomatology of the attentional disorder ADHD has been negatively associated with levels of P50 suppression, as well as its peak amplitude, and peak latency (29). Likewise, a study with adults reported that individuals with ADHD had lower average P50 suppression compared to healthy controls (30). However, others report that P50 deficits are not associated to disorders that are related to a wider distribution of attention such as ADHD or first-episode psychosis (31, 32). Similar to P50 suppression, children with ADHD have been shown to have reduced levels of PPI (33). Yet, results are scarce and do not show a definite relation given that replications of this result in adults and in adolescents were unsuccessful (30, 34, 35). Moreover, while the majority of studies have shown that gating deficits are unrelated to neuropsychological measures (30, 32, 34, 36), there are some interesting exceptions. For instance, a positive association between executive functioning and P50 suppression has been reported in patients with schizophrenia (37) and Alzheimer's disease (38). There are also reports on positive associations between executive functions and PPI (39–41). Executive functions are a neuropsychological concept that is commonly defined as those higher order cognitive functions that are important for planning and reaching one's goals (42) and have for instance been shown to be important in the performance of creative tasks (43, 44). These studies indicate that even though often left uninvestigated, sensory gating can be seen as the preliminary filtering system, before executive functions, that plays a role in creative cognition (45). There are accounts that indicate that when the environment is rich in stimuli and contains many action possibilities, this might lead to more creative ideations. That is, if individuals are able to perceive many action possibilities due to reduced sensory gating, even if these possibilities are only implicitly relevant to the task at hand, they are able to use that knowledge creatively. Additionally, less stringent filtering processes might also lead to an influx of less relevant knowledge into working memory when working on a task (46–48). Hypothetically, when more leaky attention (for instance due to reduced sensory gating) is present, chances increase that more (implicitly relevant) action possibilities will be observed and used during creative tasks. Taken together, these reports suggest that less stringent gating abilities lead to increased creativity by exerting influence on executive functions.

To date, only two EEG-studies exist in which the association between sensory gating and creativity was investigated (8, 49). Zabelina et al. reported that divergent thinking was related to increased sensory gating and that real-life creative achievements were related to reduced sensory gating in a sample of healthy adults (8). They therefore reasoned that divergent thinking depends on the ability to rapidly focus attention by restraining sensory gating. However, results may be different when there are no severe time constraints. Since reduced gating theoretically leads to a wider range of available stimuli in working memory to combine, this in turn might increase the amount of creative ideations that are generated (50, 51). This is in line with the second study that examined P50 suppression in relation to divergent and convergent measures of creativity (49). They found that while convergent thinking was positively related to reduced sensory gating, there was a negative association between the divergent thinking measure fluency and sensory gating. In addition, no relation was found with the divergent thinking measure of originality.

Thus far, not much creativity research exists that makes use of neuroscientific methods. By investigating these underlying mechanisms of behavioral concepts such as attention these methods can contribute to valuable new insights of such concepts. As such, it is increasingly accepted that a continued effort should be made to increase our knowledge by providing a more mechanistic account of processes such as attention and creativity (52, 53). Neurocognitive disorders related to attentional deficits such as schizophrenia and ADHD have been associated with both sensory gating deficits and increased creativity (54, 55). However, results also suggest that as symptom severity increases to clinical levels, potential strengths such as being more creative often disappear (54, 56, 57). Therefore, the current study aims to investigate if individual differences in sensory and sensorimotor gating that are present in the general population can explain the relationship between attention and attentional deficits such as those found in ADHD and creative thinking.

We hypothesized that higher levels of creativity as well as attentional difficulties would be related to an individual's increased responses to irrelevant stimuli, and thus lesser PPI and P50 suppression. Additionally, we exploratively examined if there was a difference in sensory- and sensorimotor gating between those children in our population that happened to have an ADHD/ADD diagnosis and typically developing children.

## Materials and Methods

All tests were performed at the Utrecht Medical Centre in The Netherlands and approved by the Faculty Ethics Review Board (FETC18-081) of the Faculty of Social and Behavioural Sciences at Utrecht University and the Medical Ethical Committee of the Utrecht Medical Centre (NWMO18-849).

### Participants

Participants were invited from a behavioral study that investigated the relation between creativity, executive functioning, and mathematics in a natural population of primary school children (*N* = 360) (50). The information letter for the behavioral study already contained information regarding the EEG study, which would be on voluntary basis. Therefore, after data collection of the behavioral part was finished, the parents of the participating children were contacted once more about their child's willingness to participate in the EEG study as well; In total, 70 of these invited 360 children were willing to do so. In order to test for potential selection bias in our current sample, we compared the group of children who participated in the behavioral study (*n* = 360) with those, who agreed to participate in the currently reported EEG study as well (*n* = 70). After Bonferroni correction for multiple testing (0.05/11), we found one significant difference: The group of children that did not participate in the EEG study showed on average higher fluency than the group that did participate (*p* = 0.003, Cohen's *d* = 0.305). No other significant group differences were found regarding age, gender, intelligence, scores on flexibility, originality, giftedness nor occurrence of dyslexia, ASD or occurrence of ADHD/ADD (*p* >0.016).

Three children were excluded based on a suspicion or diagnosis of an autism spectrum disorder, as indicated by the parents. One participant showed no identifiable P50 waveform, 30 participants did not show PPI above noise level or were unable to finish the task due to discomfort caused by the sheer intensity of the stimuli, and data from one participant was lost due to technical issues. Additionally, missing data was present for one participant on the Test for Creative Thinking-Drawing Production (58), for two participants on the mathematical creativity test, and three participants on the attentional questionnaire. Hence, 65 participants (36 boys) were included in the analyses of the P50 paradigm (Mean age = 10.77; *SD* = 0.84; Range = 9.30–12.72), of which 7 had an ADHD/ADD diagnosis or their parents had a suspicion they had ADHD/ADD and 37 participants (23 boys) were included in the analyses of the PPI paradigm (Mean age = 10.79; *SD* =0.70; Range = 9.30–12.40) of which 6 had an ADHD/ADD diagnosis or their parents had a suspicion they had ADHD/ADD.

### Procedure

Before testing, we obtained active informed consent from at least one parent. The Copenhagen Psychophysiological Testbattery (59) was assessed in a dimly lit, soundproof room; the battery took ~70 min to complete. Beforehand, a screening for possible hearing deficits was performed at 500, 1,000, and 6,000 Hz (40 dB) for which the participant was instructed to indicate if they heard the tone in their left or right ear by raising their ipsilateral hand. If participants could not hear a tone or identify its origin they were excluded from the study. Participants were instructed to sit upright but relaxed. Given the specific topic of this paper, we only focussed on the results of the sensory- and sensorimotor gating tasks, the other results will be published elsewhere.

### Behavioral Instruments

#### Test for Creative Thinking-Drawing Production (TCTDP)

To test general creativity, participants completed the TCTDP. Participants had 15 min to complete a drawing containing six figural fragments. Fourteen creativity aspects were scored to create a total score. The interrater reliability was found to be good (*r* = 0.87) and the differential reliability is high [χ^2^ = 33.45, C_(corr.)_ = 0.92] (58).

#### Mathematical Creativity Test

To test different subcomponents of divergent creativity, we administered a mathematical multiple solution test containing four questions. One question was to name multiple ways to equally divide a cake among four people (60). The other three questions were about identifying reasons why a shape did not belong to a group of shapes, why a number did not belong, and thinking of multiple ways how a calculation can start with “7” and have the answers “21” (61, 62). All questions were scored on originality (the novelty of an answer), flexibility (the different strategies or categories answers belong to), and fluency (the number of correct answers provided). The internal consistency of the task was acceptable (Cronbach's α = 0.78) (61).

#### Strength and Difficulties Questionnaire

Attentional difficulties were assessed with the SDQ, a comprehensive questionnaire filled in by parents (63). The SDQ had 25 questions with a 3-point Likert scale and was comprised of five scales. The reported internal consistency of the hyperactivity/inattention subscale is satisfactory (Cronbach's α between 0.65 and 0.88) and concurrent validity is acceptable (63, 64).

#### Intelligence

The subtest “results” from the NIO (Dutch intelligence test for educational level; Nederlandse Intelligentietest voor Onderwijsniveau) was used. During this task, participants had to indicate which of five two-dimensional shapes can be folded into a three-dimensional shape. For each assignment, a total of five points could be scored and a sumscore was created. Internal consistency for the subtest “results” was good (Cronbach's α = 0.82) (65).

### Paradigms

#### PPI and Habituation Paradigm

This paradigm took 27 min and started with the presentation of 5 min of white, background noise of 70 dB to acclimate the participant. Hereafter, during Block 1 and 3, a series of eight pulse-alone (PA) trials were presented with white noise burst of 105 dB lasting 20 ms to measure habituation. The intertrial intervals were randomized between 10 and 20 s. Block 2 also contained PA trials of which there were 10 in total that were identical to those in block 1 and 3. Additionally, block 2 also contained prepulse-pulse trials to measure PPI. In these prepulse-pulse trials, prepulses consisted of white noise bursts of either 76 or 85 dB lasting 20 ms after which a pulse (identical to a PA) followed. Intertrial intervals were randomized between 10 and 20 s. The stimulus onset asynchrony between the prepulse and pulse stimuli was either 60 or 120 ms. Hence, four types of PPI combinations were present in this paradigm: 60 ms/76 dB, 120 ms/85 dB, 60 ms/76 dB, and 120 ms/85 dB. Each prepulse-pulse combination was presented 10 times, which in combination with the 10 PA trials of block 2 leads to a total of 50 trials in the paradigm. Given the specific topic of the current paper, habituation was not investigated.

#### P50 Paradigm

To measure the P50 ERP, a standard P50 paradigm was used. Auditory stimuli were paired, short bursts of white noise of 90 dB and a duration of 1.4 ms, with a 10 s ISI and 500 ms intrapair interval. Stimuli were presented in three blocks of 40 click-pairs to combat drowsiness and boredom. Participants were instructed to count the clicks to avoid drowsiness even further (4, 36, 59). Each block lasted ~7 to 8 min.

### Data Processing

The auditory stimuli were presented by a computer running Presentation^®^ (Neurobehavioral Systems, Inc., Albany, CA, USA) software. Participants wore stereo insert headphones (Eartone ABR©1996–2008, Interacoustics A/S, USA). BioSemi^®^ hardware (BioSemi, Netherlands), containing 64 Active Two electrodes arranged according to the extended 10–20 system (66) continuously recorded a subject's electroencephalogram (EEG) and electromyogram (EMG) activity. Sampling was done in a continuous fashion. Signals were digitized at 2 kHz by a computer.

The eye-blink component of the acoustic startle response was measured by recording EMG activity from the right orbicularis oculi, for which purpose two electrodes were placed underneath the right eye of the participant. The first of these was aligned with the pupil, the other was positioned laterally. BESA software (version 6.0, MEGIS Software, Gräfelfing, Germany) was used to process the data.

#### PPI Paradigm

First, data was filtered between 25 and 250 Hz. Hereafter the highest amplitude in the time interval 20–140 ms after the startle-eliciting pulse was scored as the startle amplitude. Here, PPI was calculated as [(1 – (PP/PA)) × 100%] in which PP was the average startle amplitude to prepulse—pulse trials and PA was the average amplitude to pulse alone trials of block 2. Based on the different stimulus onsets (60 or 120 ms) and intensities (76 or 85 dB) this resulted in five outcome measures PPI 7660, PPI 76120, PPI 8560, PPI 85120, including PA.

#### P50 Paradigm

The surrogate model of BESA was used to correct for eye-blinks and -movements. If the difference between minimum and maximum amplitude exceeded 150 μV in the relevant scoring window, epochs were removed as a correction for non-paradigm-related artifacts. Hereafter, all epochs were averaged and subsequently filtered between 1 and 70 Hz. The largest through-to-peak P50 amplitudes were scored from electrode Cz, where the highest amplitude was reached, with average reference in the 35–90 ms interval after the first (conditioning, C) stimulus of the paired-click trial (67, 68). The P50 amplitude of the second (testing, T) stimulus was defined as the largest through-to-peak amplitude within the latency of the maximum P50 amplitude to the C-stimulus, ± 10 ms. P50 suppression was expressed as the ratio T/C.

### Statistical Analysis

All data was analyzed with IBM SPSS Statistics 24. The distribution of the data was determined with Kolmogorov Smirnov tests. First, the correlations between all variables were checked and possible covariates for age, gender and intelligence were identified. Second, regression analyses were performed to assess the relation between psychophysiological gating measures, creativity and attention. Third, we grouped participants based on their sensory and sensorimotor gating performance. For each of these outcome variables, two groups were created based on scores above and below the group mean. Fourth, we investigated if these groups differed on creativity and attentional measures. As such, ANCOVAs or Mann Whitney U tests were performed, depending on whether intelligence, age and gender correlated significantly with the dependent variables or not. In addition, we performed Mann Whitney U tests to examine if the children with ADHD/ADD had significantly different P50 suppression or PPI in comparison to children without ADHD/ADD.

## Results

Table 1 shows the descriptive statistics of the P50 suppression parameters, creativity, and attentional outcome measures. Table 2 shows descriptive statistics of the PPI parameters, creativity, and attentional outcome measures. The correlation matrix (see Supplementary Table 1) shows all measures that were correlated in the current study. Based on the significant correlations displayed there, we corrected for intelligence on all creativity measures. Furthermore, we corrected for gender on PPI trial types PPI 7660, PPI 8560, and PPI 85120. Based on the scatterplots between the main variables of the current study (see Supplementary Figures 1, 2) we removed two additional participants from further analyses related to the PPI parameter PPI 7660 due to extreme values (value −158 and value −2,232).

**Table 1:** Descriptive statistics of P50 suppression parameters, creativity and SDQ attention scale.

	***n***	**Mean**	**Median**	**SD**	**Minimum**	**Maximum**
Conditioning amplitude	65	1.58 μV	1.25	1.11	0.25	4.57
Testing amplitude	65	0.88 μV	0.61	0.90	0.00	3.57
T/C ratio	65	0.71	0.44	0.86	0.00	3.52
Fluency	63	15.31	13.00	7.79	1	40
Flexibility	63	7.58	8	2.28	1	13
Originality	63	1.77	1.8	0.61	0.20	3.20
TCTDP	64	22.28	20.00	10.68	4	49
SDQ Attention	62	3.74	3.5	2.52	0.00	10.00

*Creativity variables are Fluency, Flexibility, Originality, and TCTDP. P50 suppression parameters are Conditioning Amplitude, Testing Amplitude, and T/C ratio. SD, Standard Deviation*.

**Table 2:** Descriptive statistics of the PPI parameters, creativity variables, and the SDQ attention scale.

	***n***	**Mean**	**Median**	**SD**	**Minimum**	**Maximum**
Pulse alone	37	47.32 μV	38.00	26.23	22.00	113.00
PPI 7660	35	21.49%	27.00	33.52	−62.00	85.00
PPI 76120	37	30.46%	39.00	33.25	−66.00	87.00
PPI 8560	37	16.16%	26.00	42.56	−138.00	80.00
PPI 85120	37	37.76%	50.00	31.47	−43.00	89.00
Fluency	37	13.46	12.00	7.47	1.00	38.00
Flexibility	37	6.95	7.00	2.33	1.00	12.00
Originality	37	1.70	1.60	0.64	0.20	3.20
TCTDP	37	23.00	21.00	10.97	4	49
SDQ Attention	37	4.11	4.00	2.73	0.00	10.00

*Creativity variables are Fluency, Flexibility, Originality, and TCTDP. PPI parameters are Pulse alone, PPI 7660, PPI 76120, PPI 8560, and PPI 85120. SD, Standard Deviation*.

**Figure 1:**
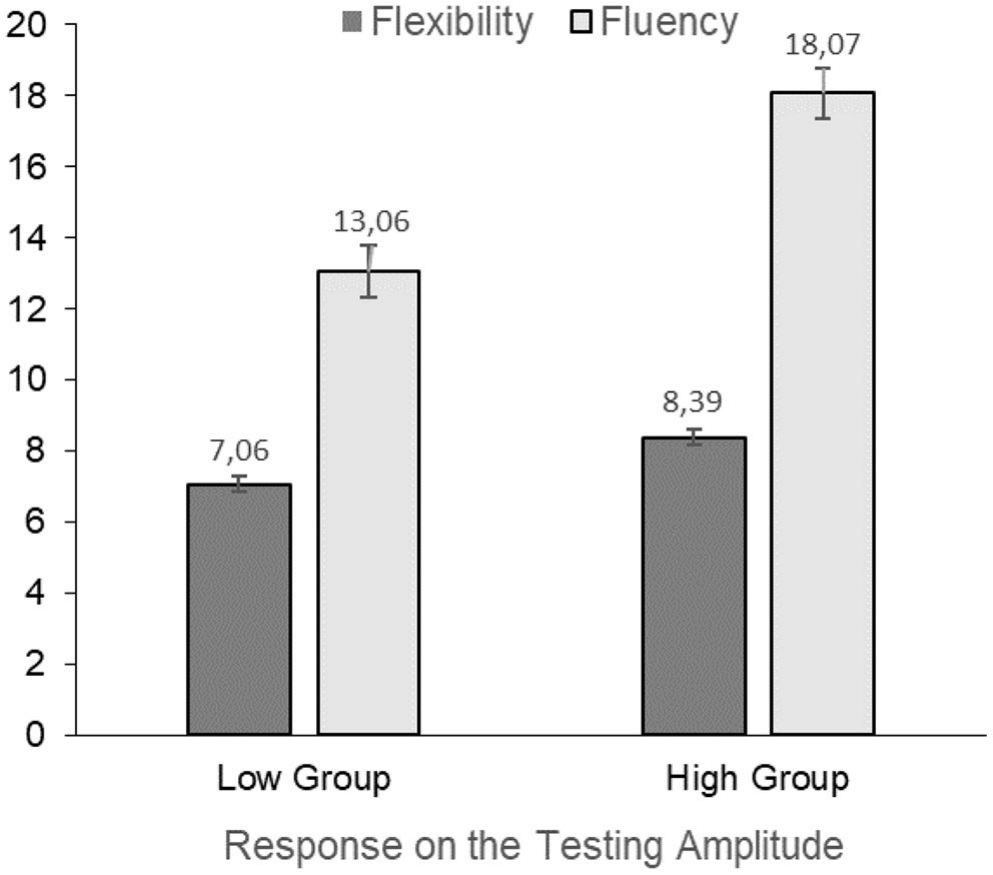
Significant difference in mathematical creativity components fluency and flexibility between the group with higher testing amplitudes and the group with lower amplitudes in the P50 suppression paradigm. The estimated marginal means and standard error of the groups displayed in the figure are corrected for intelligence.

Based on our hypothesis that the low sensory gating group would have increased creativity scores we first performed multiple regression analyses to assess the relation between the psychophysiological measures, creativity and attention, but found no significant associations between these measures, likely due to our sample size (see Supplementary Table 2 for the P50 suppression parameters and Supplementary Table 3 for the PPI parameters). To explore possible associations between these measures more thoroughly, we subsequently split the participants on the sensori(motor) gating parameters and performed ANCOVAs to test our predictions. These results can be viewed in Table 3. When split on (above and below) average conditioning amplitude we found no significant group effects on any of the creativity scores, i.e., fluency, flexibility, originality, or TCTDP. When split on (above and below) average testing amplitude a significant main effect of group was found on fluency and flexibility, indicating higher creativity scores in the group with higher testing amplitude than in the group with lower amplitudes. These significant associations are depicted in Figure 1. However, no significant main group effect was found on originality or TCTDP. Similar to the split on conditioning amplitude, when split on (above and below) average T/C ratio no significant group effects in the creativity measures were found for fluency, flexibility, originality, or TCTDP.

**Table 3:** ANCOVA statistics for P50 suppression parameters and creativity variables.

	**F ratio**	***df***	***p***	**math!!!**
**Conditioning Amplitude**				
Fluency	0.613	1.59	0.437	0.010
Flexibility	4.329	1.59	0.198	0.028
Originality	3.085	1.59	0.904	0.000
TCTDP	3.085	1.60	0.084	0.049
**Testing Amplitude**				
Fluency	6.524	1.59	0.013	0.100
Flexibility	6.556	1.59	0.013	0.100
Originality	2.600	1.59	0.112	0.042
TCTDP	0.377	1.60	0.541	0.006
**T/C Ratio**				
Fluency	0.149	1.59	0.701	0.003
Flexibility	0.787	1.59	0.787	0.013
Originality	0.008	1.59	0.928	0.000
TCTDP	0.006	1.60	0.798	0.001

*Creativity variables are Fluency, Flexibility, Originality, and TCTDP. P50 suppression parameters are Conditioning Amplitude, Testing Amplitude, and T/C ratio. SD, Standard Deviation*.

Since the SDQ attention scale did not significantly correlate with any of our covariates, Mann Whitney U tests were performed to test our prediction that the group with low sensory gating would have increased attentional difficulties. These tests showed no significant group differences when groups were split on (above or below) average conditioning amplitude (*U* = 414.50, *p* = 0.617), testing amplitude (*U* = 32.13, *p* = 0.727), or T/C ratio (*U* = 376, *p* = 0.414).

Correlational analyses between P50 measures and measures of creativity and the SDQ attention scale showed only one significant correlation: the conditioning amplitude correlated significantly positive with flexibility (*r*_s_ = 0.338, *p* = 0.007).

Next, we spilt groups above or below average PPI to test our predictions. Results can be viewed in Table 4. No significant group differences were found for any of the creativity outcome measures (i.e., fluency, flexibility, or originality, or TCTDP) when group were split on above or below average PPI parameters pulse alone amplitude, PPI 7660, PPI 76120, PPI 8560, or PPI 85120. In addition, no significant group differences were found in the SDQ attention scale when groups were split on pulse alone amplitude, PPI 7660, PPI 76120, PPI 8560, or PPI 85120 and neither did any of the PPI measures correlate significantly with any of the creativity or attentional measures (*r*_s_ < −0.238, *p* = 0.156).

**Table 4:** ANCOVA statistics for PPI parameters, creativity and SDQ attention scale.

	**F ratio**	***df***	***p***	**math!!!**
**Pulse alone**				
Fluency	0.091	1.34	0.765	0.003
Flexibility	0.337	1.33	0.565	0.010
Originality	0.032	1.34	0.860	0.001
TCTDP	0.075	1.32	0.787	0.002
SDQ Attention	0.769	1.34	0.387	0.022
**PPI 7660**				
Fluency	0.742	1.31	0.396	0.023
Flexibility	0.562	1.31	0.459	0.018
Originality	0.069	1.31	0.794	0.002
TCTDP	0.165	1.31	0.687	0.005
SDQ Attention	0.001	1.34	0.981	0.000
**PPI 76120**				
Fluency	1.013	1.34	0.321	0.029
Flexibility	2.889	1.34	0.098	0.078
Originality	0.014	1.34	0.905	0.000
TCTDP	0.006	1.34	0.940	0.000
SDQ Attention	0.037	1.34	0.848	0.001
**PPI 8560**				
Fluency	2.605	1.33	0.116	0.073
Flexibility	0.542	1.33	0.467	0.016
Originality	0.635	1.33	0.431	0.019
TCTDP	4.028	1.33	0.053	0.109
SDQ Attention	3.941	1.34	0.055	0.104
**PPI 85120**				
Fluency	0.598	1.33	0.445	0.018
Flexibility	1.974	1.33	0.169	0.056
Originality	0.711	1.33	0.405	0.021
TCTDP	3.435	1.33	0.073	0.094
SDQ Attention	0.865	1.34	0.359	0.025

*Creativity variables are Fluency, Flexibility, Originality, and TCTDP. PPI parameters are Pulse alone, PPI 7660, PPI 76120, PPI 8560, and PPI 85120*.

To inspect if children with (a suspicion of) ADHD/ADD differed in their P50 suppression or PPI in comparison to children without ADHD/ADD we performed Mann Whitney U tests. Descriptive statistics for the ADHD/ADD group and non-ADHD/ADD group are displayed in Table 5. These tests showed no significant group differences on the conditioning amplitude (*U* = 147.5, *p* = 0.289), testing amplitude (*U* = 188, *p* = 0.861) or T/C ratio (*U* = 166.5, *p* = 0.517), PPI 7660 condition (*U* = 49.00, *p* = 0.220), PPI 76120 condition (*U* = 152.5, *p* = 0.535), PPI 8560 condition (*U* = 162.5, *p* = 0.702), or the PPI 85120 condition (*U* = 157.5, *p* = 0.616).

**Table 5:** Descriptive statistics of the ADHD/ADD group and non-ADHD/ADD group concerning psychophysiological measures.

	***n***	**Mean**	**Median**	**Standard deviation**	**Minimum**	**Maximum**
**ADHD/ADD group**				
Conditioning amplitude	7	2.38	2.38	0.87	1.76	2.99
Testing amplitude	7	1.01	1.01	0.99	0.31	1.71
T/C ratio	7	0.37	0.37	0.28	0.18	0.57
Pulse alone	6	60.83	61.50	34.90	24.00	98.00
PPI 7660	5	8.00	4.00	15.33	−7.00	27.00
PPI 76120	6	33.17	30.50	26.71	6.00	71.00
PPI 8560	6	−20.33	−12.50	66.83	−138.00	52.00
PPI 85120	6	15.83	6.00	38.52	−29.00	70.00
**Non-ADHD/ADD group**				
Conditioning amplitude	56	1.61	1.19	1.15	0.25	4.57
Testing amplitude	56	0.88	0.62	0.91	0.00	3.57
T/C ratio	56	0.72	0.41	0.90	0.00	3.52
Pulse alone	31	44.71	38.00	24.06	22.00	113.00
PPI 7660	30	23.73	32.00	35.34	−62.00	85.00
PPI 76120	31	29.94	39.00	34.73	−66.00	87.00
PPI 8560	31	23.23	29.00	33.34	−62.00	80.00
PPI 85120	31	42.00	52.00	28.74	−43.00	89.00

## Discussion

The current study found that children with a higher P50 amplitude in response to testing stimuli in the P50 suppression paradigm had significantly higher scores on the creative measures of fluency and flexibility. No other significant differences between high and low scoring children on any of the remaining measures of sensory- or sensorimotor gating were found on other creativity measures, scores on attentional difficulties, or having an ADHD diagnosis or not.

Contrary to a previous report on a healthy adult sample that found higher creativity scores related to higher sensory gating as measured by the T/C ratio (8), we found that the children in our study with above group average responses to testing stimuli of the P50 suppression paradigm scored higher on creative fluency and flexibility (however, please note that no significant differences were observed for the T/C ratio or conditioning amplitude). Zabelina et al. (8) attributed their result to the 3 min time constraint and emphasize on the quantity of responses during their divergent thinking task. Perhaps this increased the focus on timing, resulting in less creative quality of responses (69). In comparison, the time limit of the divergent thinking task in our study was 25 minutes, which may have led to benefits of a reduced capacity for sensory gating. Alternatively, the differences between the results could also be explained by the fact that Zabelina et al. (8) reported on healthy adults, while we report on a naturalistic sample of primary school children.

We found no evidence for an association between increased originality and reduced sensory gating. It is likely that participants who experience less bottom-up control will think of more answers because such a state of dispersed attention facilitates idea generation (70). Likewise, the conditioning P50 amplitude has been reported to negatively relate to verbal fluency before (36), indicating that individuals who respond more intensely to conditioning stimuli score higher on verbal fluency when confronted with distractions. Hence, distracting, seemingly irrelevant stimuli may contain relevant information for tasks that require multiple solutions. In comparison, originality is more akin to convergent processes because it is based on finding a solution that is most optimal or novel (71). This process is different from the quantitative nature of fluency and flexibility. The ability to produce many or different responses may indeed benefit from attenuated sensory and sensorimotor gating because it is related to the preparation and incubation stage of creativity, which focusses on idea generation and assessing all possibilities (72). The second two stages are more convergent; assessment of ideas takes place whereafter the optimal response is selected. When selecting the most novel and original response, quality assessment takes place, incubation, originality and comparison take time, and top-down control and sensory gating might have to be more engaged (70, 73).

Although our results appear to contradict the often-cited positive association between executive functions and creativity (43, 44, 50), they may add to this line of research by showing that sensory and sensorimotor gating are differently related to creativity in comparison to executive functions. Speculatively, alternative protective mechanisms, such as increased working memory, well-developed executive functions or increased intelligence, in individuals with reduced sensory gating may allow them to benefit from their reduced filtering capacity during creative tasks. For instance, by allowing more stimuli to pass through the filter at an early stage in the attentional process which can be sufficiently processed thanks to well-developed executive functions and other protective mechanisms at later stages. However, if an individual suffers from substantial sensory gating deficits and has insufficient protective mechanisms to compensate for these deficits, then the outcome might still perhaps be increased creativity, but likely also accompanied by detrimental psychopathology (74, 75).

We found no association between attention or ADHD diagnosis, assessed by a parent questionnaire, and sensory or sensorimotor gating. Perhaps attentional difficulties do not originate from a general gating deficit but rather from a specific issue with controlled attention (76, 77). For instance, a sample of 10–12-year-olds with ADHD did not show any differences in PPI during a passive listening task but when participants had to actively ignore the stimuli, an attenuated PPI response was observed (78). Furthermore, adults with ADHD had a lower PPI response compared to healthy controls when task instructions were to attend to the stimuli in the paradigm, but no group differences were found during the passive listening condition (77). It is recommended that future studies compare both active and passive paradigms to detect possible differences in both creativity and attention. Indeed, it has been suggested that behaviourally observed attentional difficulties, as assessed in the current study, are more related to top-down attentional control deficiencies (79). Furthermore, while our attentional subscale of the behavioral questionnaire did not show a relation to sensory- and sensorimotor gating, there are indications that performance tests of attention do relate to P50 gating (25). Therefore, a more thorough investigation of different attentional measures and their relation to sensory- and sensorimotor gating is warranted. In addition, the hypothesis that creative individuals can flexibly alter their inhibitory and attentional processes based on task demands also fits well with this idea (2, 80).

By investigating different psychophysiological measures, our study further refined scientific knowledge about the often cited distractibility of creative individuals (9, 81). Results may help children that are easily distracted by motivating them in aspects of creativity and self-efficacy (82). However, the current study is not without its limitations. For instance, although we measured creativity in different ways, we did not include a specific convergent thinking task. Moreover, we did not collect data on parental mental history of the participating children. As a result, we cannot be sure that all parents were completely free of a psychiatric disorder, which may have influenced our data given that there are reports showing that offspring of patients with schizophrenia show less P50 suppression (83).

The sample size in the PPI analyses was rather small and the children that were unable to complete the PPI paradigm due to the stimulus intensity may all have had attenuated sensory gating. Therefore, we might have lost data from an important subgroup.

In conclusion, this study shows evidence that although sensory- and sensorimotor gating and attentional difficulties are unrelated, creativity and quantitative measures of divergent thinking such as fluency and flexibility, are at least indirectly related to decreased sensory, but not sensorimotor gating. In addition, lower sensorimotor gating seems to benefit tasks related to general creativity such as measured by our creative drawing task. Further research is required to examine if this relation is generalizable to other age groups, creativity measures, and measures of sensory gating.

## Data Availability Statement

The raw data supporting the conclusions of this article will be made available by the authors, without undue reservation.

## Ethics Statement

The studies involving human participants were reviewed and approved by the Faculty Ethics Review Board (FETC18-081) of the Faculty of Social and Behavioural Sciences at Utrecht University and the Medical Ethical Committee of the Utrecht Medical Centre (NWMO18-849). Written informed consent to participate in this study was provided by the participants' legal guardian/next of kin.

## Author Contributions

EK, BO, JV, and MS conceptualized the research. MS collected the data and wrote the original draft and edited the paper. MS and BO processed and analyzed the data. EK, JV, and BO critically reviewed the paper. All authors contributed to the article and approved the submitted version.

## Conflict of Interest

The authors declare that the research was conducted in the absence of any commercial or financial relationships that could be construed as a potential conflict of interest.

## Publisher's Note

All claims expressed in this article are solely those of the authors and do not necessarily represent those of their affiliated organizations, or those of the publisher, the editors and the reviewers. Any product that may be evaluated in this article, or claim that may be made by its manufacturer, is not guaranteed or endorsed by the publisher.
