# Supplementary material for: Prepulse Inhibition and P50 Suppression in Relation to Creativity and Attention: Dispersed Attention Beneficial to Quantitative but Not Qualitative Measures of Divergent Thinking
Source: Front Psychiatry. 2022 Jun 9;13:875398. doi: 10.3389/fpsyt.2022.875398 (PMC9218263; doi:10.3389/fpsyt.2022.875398)
Supplement: Supplementary file 1 [file Data_Sheet_1.PDF]

Table 1.

Correlation matrix between the creativity measures, SDQ attentional scale, intelligence, age, gender, and psychophysiological gating variables.

			1.	2.	3.	4.	5.	6.	7.	8.	9.	10.	11.	12.	13.	14.	15.
1.	C Amplitude	r_s															
2.	T Amplitude	r_s	**.429*** **														
3.	T/C ratio	r_s	-.101	**.821*** **													
4.	PPI 7660	r_s	-.041	-.257	-.221												
5.	PPI 76120	r_s	-.063	-.207	-.121	**.542** **											
6.	PPI 8560	r_s	-.128	-.165	-.102	**.720*** **	**.522** **										
7.	PPI 85120	r_s	.031	-.241	-.281	.399	**.657*** **	**.583*** **									
8.	Pulse Alone	r_s	.059	-.123	-.219	.031	.111	.055	.310								
9.	SDQ Attention	r_s	-.138	.005	.086	-.084	.069	-.228	-.146	-.150							
10.	TCTDP	r_s	-.069	-.156	-.148	-.032	.019	-.174	-.119	.014	-.008						
11.	Fluency	r_s	.227	.083	-.027	-.091	-.236	-.238	-.153	-.153	-.315	.311					
12.	Flexibility	r_s	.338**	.170	.017	-.039	-.237	-.123	-.136	-.095	**-.492*** **	.151	**.814*** **				
13.	Originality	r_s	.196	.011	-.084	-.026	-.039	-.168	-.177	-.163	-.301	.210	**.680*** **	**.697*** **			
14.	Gender	r_s	.065	.140	.079	**-.560*** **	-.329	**-.525** **	**-.371* **	-.272	-.220	-.141	.156	.158	.233		
15.	Age	r_s	.222	.015	-.074	-.139	.158	-.251	.065	-.122	.223	.094	.084	.077	.288	.118	
16.	Intelligence	r_s	.049	-.180	-.250	-.012	-.041	-.161	.001	-.034	-.122	**.458*** **	**.552*** **	**.426*** **	**.324** **	-.064	.132

Note. Values that were still significant after the Benjamini and Hochberg FDR correction have been made bold and underlined.

* $p < .05$. ** $p < .01$.*** $p < .001$.

Table 2.

Results of multiple regression analyses of P50 suppression parameters and creativity variables.

	B	Beta	*SE*	*t*	*p*
Conditioning Amplitude					
Fluency *	1.342	.192	.794	1.689	.097
Flexibility *	.427	.207	.236	1.807	.076
Originality *	.080	.146	.067	1.189	.239
TCTDP *	-1.372	-.142	1.151	-1.192	.238
Testing Amplitude					
Fluency *	1.626	.191	.968	1.679	.098
Flexibility *	.467	.186	.289	1.615	.112
Originality *	.065	.097	.083	.785	.435
TCTDP *	-.190	-.016	1.425	-.133	.894
T/C Ratio					
Fluency **	.536	.055	1.112	.482	.632
Flexibility *	.250	.088	.340	.735	.466
Originality *	-.002	-.003	.096	-.021	.984
TCTDP *	-.206	-.015	1.634	-.126	.900

Note. Creativity variables = Fluency, Flexibility, Originality, and TCTDP. P50 suppression parameters = Conditioning Amplitude, Testing Amplitude, and T/C ratio.

* This regression model showed a significant relation with the coviariate intelligence

** This regression model showed a significant relation with the coviariates intelligence and gender

Table 3.

Results of multiple regression analyses of PPI parameters x creativity variables and attentional SDQ scale.

	B	Beta	*SE*	*t*	*p*
Pulse Alone					
Fluency **	-.062	-.218	.041	-1.511	.140
Flexibility *	-.005	-.057	.014	-.364	.718
Originality	-.002	-.067	.004	-.395	.695
TCTDP	.009	.022	.071	.128	.899
SDQ Attention	-.005	-.044	.018	-.261	.796
PPI 7660					
Fluency *	-.048	-.214	.035	-1.382	.177
Flexibility *	-.008	-.113	.011	-.699	.490
Originality	.001	.058	.003	.334	.740
TCTDP *	-.004	-.015	.048	-.094	.926
SDQ Attention	-.002	-.026	.014	-.148	.883
PPI 76120					
Fluency **	-.006	-.029	.033	-.196	.846
Flexibility *	-.016	-.230	.011	-1.530	.135
Originality	.001	062	.003	.369	.714
TCTDP	.005	.014	.056	.082	.935
SDQ Attention	.009	.116	.014	.688	.496
PPI 8560					
Fluency **	-.003	-.015	.029	-.087	.931
Flexibility *	-.006	-.116	.009	-.748	.460
Originality	.000	-.029	.003	-.172	.864

	TCTDP	-.037	-.144	.043	-.862	.394
	SDQ Attention	-.010	-.156	.011	-.936	.356
PPI 85120						
	Fluency *	-.054	-.228	.035	-1.530	.135
	Flexibility *	-.015	-.201	.011	-1.314	.198
	Originality	-.003	-.126	.003	-.749	.459
	TCTDP	-.060	-.171	.058	-1.025	.312
	SDQ Attention	-.011	-.121	.015	-.724	.474

Note. Creativity variables = Fluency, Flexibility, Originality, and TCTDP. PPI parameters = Pulse alone, PPI 7660, PPI 76120, PPI 8560, and PPI 85120.

* This regression model showed a significant relation with the coviariate intelligence

** This regression model showed a significant relation with the coviariates intelligence and gender

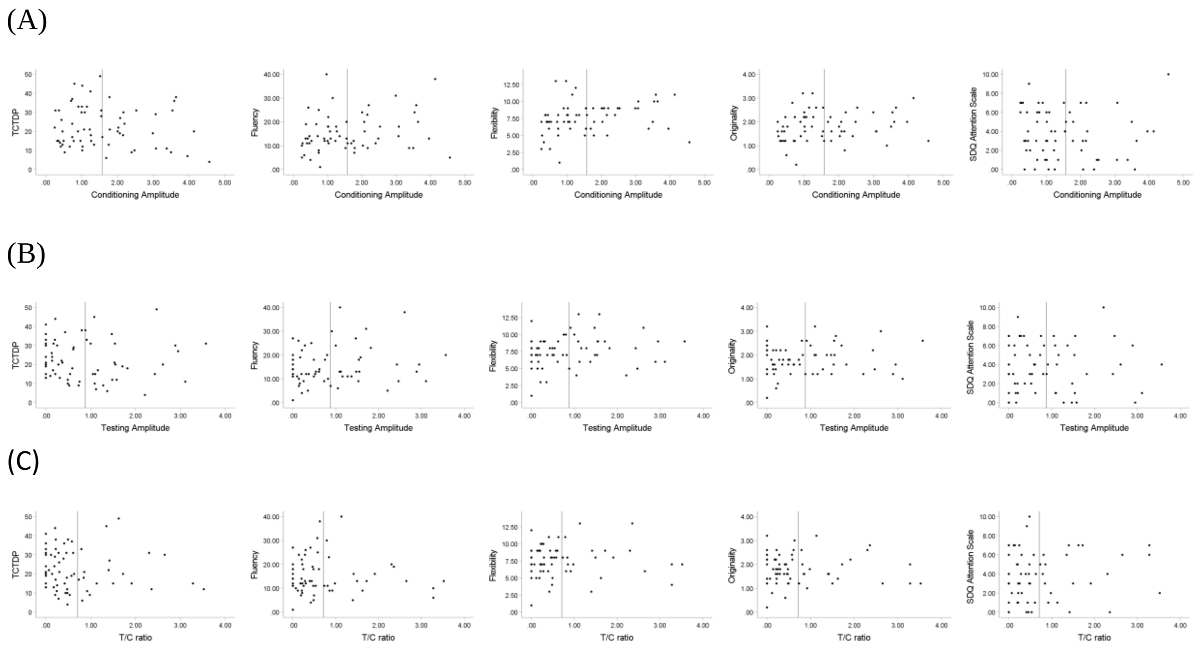

Figure 1. Scatterplots between P50 parameters, creativity variables and SDQ attention scale.

Note. Creativity variables are Fluency, Flexibility, Originality, and TCTDP. P50 suppression parameters are Conditioning Amplitude (A), Testing Amplitude (B), and T/C ratio (C). The line on the X-axis represents the psychophysiological mean value.

(A)

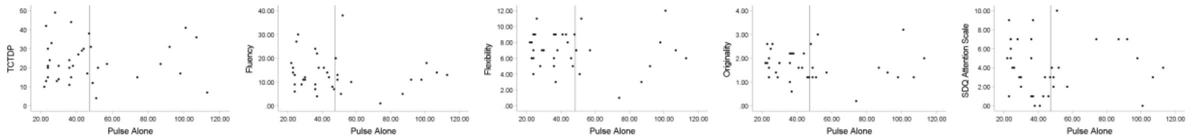

(B)

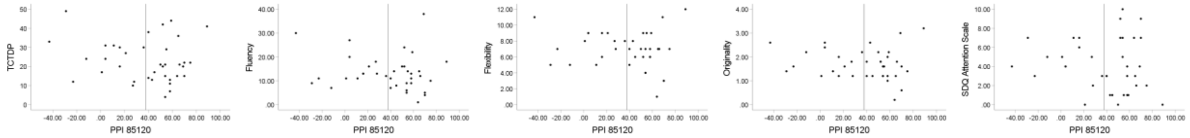

(C)

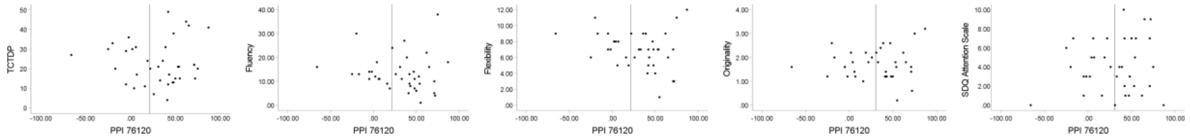

(D)

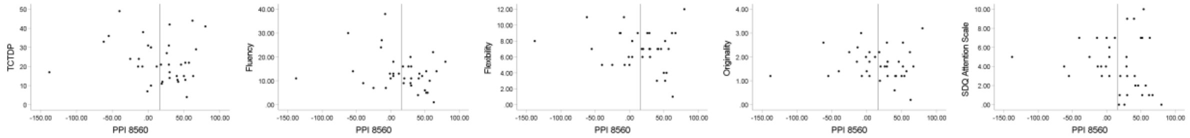

(E)

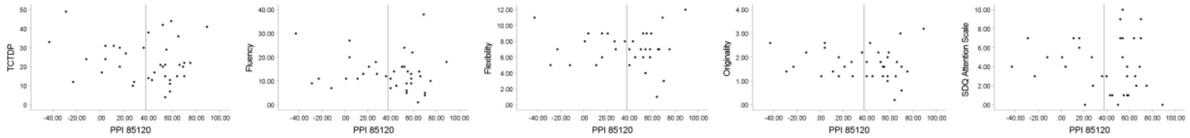

Figure 2. Scatterplots between PPI parameters, creativity and SDQ attention scale.

Note. Creativity variables are Fluency, Flexibility, Originality, and TCTDP. PPI parameters are Pulse Alone (A), PPI 7660 (B), PPI 76120 (C), PPI 8560 (D), and PPI 85120 (E). Two extreme outliers were removed in the graphs of the associations with PP7660 (see manuscript for more details). The line on X-axis represents the psychophysiological mean value.
